# ESAT: Environmental Source Apportionment Toolkit Python package

**DOI:** 10.21105/joss.07316

**Published:** 2024-12-10

**Authors:** Deron Smith, Michael Cyterski, John M Johnston, Kurt Wolfe, Rajbir Parmar

**Affiliations:** 1 United States Environmental Protection Agency, Office of Research and Development, Center for Environmental Measurement and Modeling

## Summary

Source apportionment is an important tool in environmental science where sample or sensor data are often the product of many, often unknown, contributing sources. Source apportionment is used to understand the relative contributions of air sources (Bhandari et al., 2022) like vehicle emissions, industrial activities, or dust; as well as particulate matter pollution and to identify relative contributions of point sources and non-point sources in water bodies such as lakes, rivers, and estuaries (Jiang et al., 2019; Mamun & An, 2021). Using non-negative matrix factorization (NMF), source apportionment models estimate potential source profiles and contributions providing a cost-efficient method for further strategic data collection or modeling.

Environmental Source Apportionment Toolkit (ESAT) is an open-source Python package that provides a flexible and transparent workflow for source apportionment using NMF algorithms, developed to replace the EPA’s Positive Matrix Factorization version 5 (PMF5) application (EPA, 2014; Pentti Paatero, 1999). ESAT recreates the source apportionment workflow of PMF5 including pre- and post-processing analytical tools, batch modeling, uncertainty estimations and customized constraints. ESAT offers a simulator for generating datasets from synthetic profiles and contributions, allowing for model output evaluation. The synthetic profiles can be randomly generated, use a pre-defined set of profiles, or be a combination of the two. The random synthetic contributions can follow specified curves and value ranges. By running ESAT using the synthetic datasets, users are able to accurately assess ESAT’s ability to find a solution that recreates the original synthetic profiles and contributions.

### Statement of Need

The EPA’s PMF5, released in 2014, provides a widely-used source apportionment modeling and analysis workflow that is no longer supported and relies on the proprietary Multilinear Engine v2 (ME2). ESAT has been developed as a replacement to PMF5, and has been designed for increased flexibility, documentation and transparency.

The Python API and CLI of ESAT provides a programmatic interface that can recreate the PMF5 workflow. The matrix factorization algorithms in ESAT have been written in Rust for runtime optimization. The ESAT API and CLI provides a flexible way to create source apportionment workflows and novel research applications. ESAT was developed for environmental research, though it’s not limited to that domain, as matrix factorization is used in many different fields.

### Algorithms

Source apportionment algorithms use a loss function to quantify the difference between the input data matrix (V) and the product of a factor contribution matrix (W) and a factor profile matrix (H), weighted by an uncertainty matrix (U) (Pentti Paatero & Tapper, 1994). The goal is to find factor matrices that best reproduce the input matrix, while constraining all, or most of, the factor elements to be non-negative. The solution, W and H, can be used to calculate the residuals and overall model loss. ESAT has two NMF algorithms for updating the profile and contribution matrices: least-squares NMF (LS-NMF) (Wang et al., 2006) and weighted-semi NMF (WS-NMF) (Ding et al., 2008; Melo & Wainer, 2012).

The loss function used in ESAT, and PMF5, is a variation of squared-error loss, where data uncertainty is taken into consideration (both in the loss function and in the matrix update equations):

$$Q=\sum_{i=1}^{n}\sum_{j=1}^{m}{\left[\frac{V_{ij}-\sum_{k=1}^{K}W_{ik}H_{kj}}{U_{ij}}\right]}^{2}$$

here V is the input data matrix of features (columns=M) by samples (rows=N), U is the uncertainty matrix of the input data matrix, W is the factor contribution matrix of samples by factors=K, H is the factor profile of factors by features.

The ESAT versions of NMF algorithms convert the uncertainty U into weights defined as $Uw=\frac{1}{U^{2}}$. The update equations for LS-NMF then become:

$$H_{t+1}=H_{t}\circ \frac{W_{t}(V\circ Uw)}{W_{t}\left(\left(W_{t}H_{t}\right)\circ Uw\right)}$$

$$W_{t+1}=W_{t}\circ \frac{(V\circ Uw)H_{t+1}}{\left(\left(W_{t}H_{t+1}\right)\circ Uw\right)H_{t+1}}$$

while the update equations for WS-NMF:

$$W_{t+1,i}={\left(H^{T}Uw_{i}^{d}H\right)}^{-1}\left(H^{T}Uw_{i}^{d}V_{i}\right)$$

$$H_{t+1,i}=H_{t,i}\sqrt{\frac{{\left(\left(V^{T}Uw\right)W_{t+1}\right)}_{i}^{+}+{\left[H_{t}{\left(W_{t+1}^{T}UwW\right)}^{-}\right]}_{i}}{{\left(\left(V^{T}Uw\right)W_{t+1}\right)}_{i}^{-}+{\left[H_{t}{\left(W_{t+1}^{T}UwW\right)}^{+}\right]}_{i}}}$$

where $W^{-}=\frac{\left(|W|-W\right)}{2.0}$ and $W^{+}=\frac{\left(|W|+W\right)}{2.0}$.

### Error Estimation

An important part of the source apportionment workflow is quantifying potential model error. ESAT offers the error estimation methods that were developed and made available in PMF5 (Brown et al., 2015; P. Paatero et al., 2014).

The displacement method (DISP) determines the amount that a source profile feature, a single value in the H matrix, must increase and decrease to cause targeted changes to the loss value. One or more features can be selected in the DISP uncertainty analysis. The bootstrap method (BS) uses block bootstrap resampling with replacement to create datasets with the original dimensions of the input, where the order of the samples has been modified in blocks of a specified size. The BS method then calculates a new model from the bootstrap dataset, and original initialization, to evaluate how the profiles and concentrations change as a result of sample reordering. The bootstrap-displacement method (BS-DISP) is the combination of the two techniques, where DISP is run for each bootstrap model on one or more features.

These error estimation methods address different uncertainty aspects: DISP targets rotational uncertainty, BS addresses random errors and sample variability, and BS-DISP provides the most comprehensive understanding of how the uncertainty impacts a source apportionment solution.
